# Utilizing PacBio Iso-Seq for Novel Transcript and Gene Discovery of Abiotic Stress Responses in *Oryza sativa* L.

**DOI:** 10.3390/ijms21218148

**Published:** 2020-10-31

**Authors:** Stephanie Schaarschmidt, Axel Fischer, Lovely Mae F. Lawas, Rejbana Alam, Endang M. Septiningsih, Julia Bailey-Serres, S. V. Krishna Jagadish, Bruno Huettel, Dirk K. Hincha, Ellen Zuther

**Affiliations:** 1 Max Planck Institute of Molecular Plant Physiology, Am Mühlenberg 1, 14476 Potsdam, Germany; afischer@mpimp-golm.mpg.de (A.F.); lfl0008@auburn.edu (L.M.F.L.); Hincha@mpimp-golm.mpg.de (D.K.H.); 2 Department of Biological Sciences, Auburn University, Auburn, AL 36849, USA; 3 Center for Plant Cell Biology, Department of Botany and Plant Sciences, University of California Riverside, Riverside, CA 92521, USA; ralam001@ucr.edu (R.A.); serres@ucr.edu (J.B.-S.); 4 Department of Soil and Crop Sciences, Texas A&M University, College Station, TX 77843, USA; eseptiningsih@tamu.edu; 5 International Rice Research Institute, DAPO Box 7777, Metro Manila 1301, Philippines; kjagadish@ksu.edu; 6 Department of Agronomy, Kansas State University, Manhattan, KS 66506, USA; 7 Max Planck Genome Centre Cologne, Carl-von-Linné-Weg 10, 50829 Cologne, Germany; huettel@mpipz.mpg.de

**Keywords:** dehydrins, natural genetic variation, PacBio Sequel, RNA-Seq, SMRT sequencing, de novo reference transcriptomes, rice

## Abstract

The wide natural variation present in rice is an important source of genes to facilitate stress tolerance breeding. However, identification of candidate genes from RNA-Seq studies is hampered by the lack of high-quality genome assemblies for the most stress tolerant cultivars. A more targeted solution is the reconstruction of transcriptomes to provide templates to map RNA-seq reads. Here, we sequenced transcriptomes of ten rice cultivars of three subspecies on the PacBio Sequel platform. RNA was isolated from different organs of plants grown under control and abiotic stress conditions in different environments. Reconstructed de novo reference transcriptomes resulted in 37,500 to 54,600 plant-specific high-quality isoforms per cultivar. Isoforms were collapsed to reduce sequence redundancy and evaluated, e.g., for protein completeness (BUSCO). About 40% of all identified transcripts were novel isoforms compared to the Nipponbare reference transcriptome. For the drought/heat tolerant *aus* cultivar N22, 56 differentially expressed genes in developing seeds were identified at combined heat and drought in the field. The newly generated rice transcriptomes are useful to identify candidate genes for stress tolerance breeding not present in the reference transcriptomes/genomes. In addition, our approach provides a cost-effective alternative to genome sequencing for identification of candidate genes in highly stress tolerant genotypes.

## 1. Introduction

Global climate change is causing an increase in the severity and frequency of abiotic stress conditions such as heat, drought and high night temperatures that all have a strong negative impact on crop yield [1,2,3,4,5]. In combination with the increasing world population, plant breeders face the challenging task of developing new cultivars that produce higher yield, with enhanced quality and accompanied by reduced environmental footprints [6]. Rice (*Oryza sativa* L.) is the main source of calories for more than half of the world’s population, especially for the poorest in Asia [7]. As an important reservoir for genes that may be used for crop improvement, the wide natural genetic diversity within the species and its wild relatives, which is preserved in more than 230,000 rice germplasm accessions, maintained in gene banks worldwide [8], is an invaluable resource.

While almost 80% of rice cultivation in the world is based on varieties of the *indica* subspecies [9], the current gold standard genome assembly and annotation is derived from the cultivar Nipponbare of the *japonica* subspecies [10]. Due to the lack of proper genome assemblies, studies of cultivars from different *O. sativa* subspecies have largely been based on this reference genome. For instance, the sequences obtained in the 3000 Rice Genomes Project [8] were mapped against the Nipponbare genome, excluding all sequences that could not be mapped to this reference [11]. This may have led to the loss of genetic information that is specific to the non-*japonica* subspecies. However, more recently the genomes of cultivars belonging to additional *O. sativa* subspecies have been sequenced, such as *indica* (e.g., the cultivars Shuhui498 (R498 genome; [12]), Zhenshan 97, and Minghui 63 [13]), or *aus* (e.g., the cultivars Kasalath [14] and N22 [6]), although the degree of completeness and annotation remains variable. In particular, the *aus* subspecies (addressed as own phylogenetic group more closely related to the *indica* than *japonica* subspecies [15]) has been a valuable source of genes underlying traits for disease resistance [16], tolerance to phosphate starvation [17], submergence [18], deep water growth [19], anaerobic germination [20,21] and drought [22]. For example, the phosphate-starvation tolerance gene *OsPSTOL1*, the deepwater escape genes *OsSNORKEL1/2* and the submergence tolerance gene *OsSUB1A* were identified in the genomes of *aus* cultivars. Significantly, these genes are absent in the genome sequence of the *japonica* reference cultivar Nipponbare.

During the last years, RNA sequencing (in particular Illumina-based short-read RNA-Seq) has emerged as a powerful tool for analyzing transcriptomes to identify genes that show differential expression between unstressed control and various environmental stress conditions. However, the determination of transcript levels from RNA-Seq data requires reference genome or transcriptome sequences for read mapping and annotation. In rice, the identification of differentially expressed genes and transcript isoforms is determined by the reference genome [23]. Obviously, the expression data of any gene that is not represented in the reference genome/transcriptome will be lost from the analysis. This could be particularly relevant when investigating stress-tolerant exotic cultivars, land races or wild rice species, as they may contain tolerance genes not present in the reference cultivar Nipponbare. This would then severely limit the possibility to identify novel candidate genes that can support crop improvement programs.

An obvious solution to this problem would be the sequencing, assembly and annotation of the required genomes. However, this is still comparatively expensive and time-consuming. Here, we have explored a more targeted approach of sequencing and reconstructing partial transcriptomes of rice cultivars from three different subspecies that can be used as references to map RNA-Seq reads from abiotic stress experiments. For this purpose, we have used Pacific Bioscience (PacBio) Single-Molecule Real-Time (SMRT) long-read sequencing technology isoform sequencing (Iso-Seq), belonging to a new generation of sequencing methods that provide full-length transcript sequences with high throughput [24]. It thus offers the ability to sequence transcriptomes without the need for an assembly based on an existing reference genome and to discover novel transcripts and genes in stress-tolerant “exotic” genotypes. Moreover, this approach already has been successfully applied to explore and extend existing plant transcriptomes and annotations for example in sorghum [25], wheat [26,27], sugarcane [28], wild cotton [29], different panicoid grass species [30] and alfalfa [31].

## 2. Results

### 2.1. De Novo Reconstruction of Transcriptomes

We selected ten rice (*Oryza sativa* L.) cultivars of the subspecies *aus* (Dular, N22), *indica* (Anjali, IR6226-42-6-2, IR64, IR72) and *japonica* (CT9993-5-10-1M, M202, Moroberekan, Nipponbare) for this study that we have used in previous stress experiments [32,33,34,35,36]. RNA was isolated from different organs and tissues of plants grown under various control and stress conditions in climate chambers, net-houses and in the field (Table 1 and Supplementary File S1). It should be stressed that we did not aim to obtain (near) complete transcriptomes, but rather to assemble targeted partial transcriptomes with relevance to the RNA-Seq analysis of these stress treatments.

Pooled RNA samples were sequenced on the PacBio Sequel I platform on two or three SMRT cells per cultivar (Table 2). The raw data have been deposited at the NCBI’s Sequence Read Archive (SRA) [37] under the BioProject number PRJNA640670 and are freely available. In total, between 15.49 and 24.51 gigabases (GB) of sequences were obtained for the different cultivars. Sequence raw data was processed with the software IsoSeq3 using the steps ccs and lima, resulting in between 460,340 and 736,747 full-length non-chimeric reads (FLNC, containing 5′ primer, 3′ primer and poly(A) tail) for the combined SMRT cells per cultivar. After the IsoSeq3 cluster and polish steps, between 37,951 and 54,684 high-quality (HQ), as well as between 1233 and 2170 low-quality (LQ) sequences were obtained. Possible sequence contaminations by non-plant organisms were identified by alignment against the NCBI nucleotide database using blastn [38] (E ≤ 1 × 10^−10^). Isoforms without a significant hit were aligned against the NCBI protein database using blastx [38] (E ≤ 1 × 10^−10^). All sequences that showed no significant similarity to sequences from the *Viridiplantae* (green plants) family were removed, resulting in between 37,535 and 54,594 HQ full-length transcripts for further analysis (Table 2).

It has been shown for the previous PacBio sequencing platform (RSII) that correcting long reads using corresponding RNA-Seq data could lead to an increased number of HQ sequences [25,27,28,31]. This was necessary because of a relatively high rate of LQ sequences with insertions and deletions (InDels). However, the newer PacBio Sequel platform produces a higher sequencing output compared to the RSII, including a higher number of HQ and a lower number of LQ sequences [39] which we have also seen in our own data when comparing it to previous RSII studies [25,27]. To evaluate whether InDels could be a problem in our data set, we mapped all uncorrected HQ transcripts with minimap2 against the genome sequences of the corresponding subspecies. The number of InDels was extracted from the cigar string of the alignment files (Supplementary File S2). The analysis indicated that the uncorrected sequences showed only a small fraction of InDels (between 0.08% and 0.14%). Because of this low frequency of InDels and the low number of LQ sequences (Table 2), further data analysis was performed without error correction and excluding LQ transcripts.

### 2.2. Collapsing Redundant Isoforms

During library preparation, 5′ RNA degradation products can be formed and are subsequently sequenced. These degraded products have the same exonic structure but lack some 5′ sequence information and hence yield redundant isoforms that are not associated with technical bias or biological context. To tackle the problem, three different approaches to collapse redundant isoform models were tested, namely cogent, cDNA cupcake and TAMA. While cDNA cupcake and TAMA perform collapsing based on a reference genome sequence, cogent can be used without a reference sequence. Instead, it reconstructs a coding genome based on the PacBio sequences and maps the same sequences back to the reconstructed genome. Based on this mapping, it then collapses the redundant isoforms using the cDNA cupcake algorithm. For TAMA and cDNA cupcake, transcripts were mapped against the respective *O. sativa* subspecies genome sequences using minimap2. Only a small number of transcripts were not mapped by these approaches (Table 3). With cogent, a much larger number of transcripts (5441 to 7979) could not be mapped back against the respective reconstructed coding genomes. In general, all three tools reduced the number of isoforms strongly, by 47.6% (cDNA cupcake, Nipponbare) to 68.3% (cogent, Dular) after collapsing.

**Figure 1:**
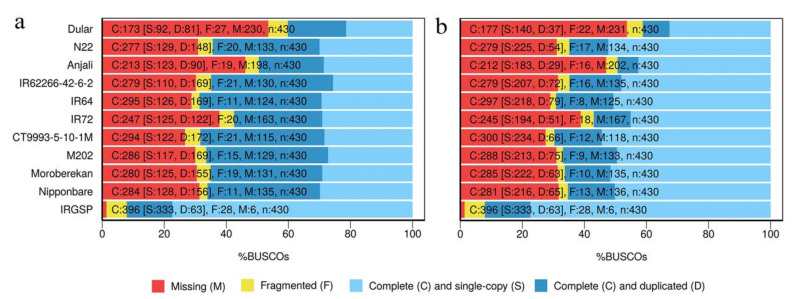
BUSCO assessment analysis of uncollapsed (**a**) and collapsed (**b**) transcripts. Results of collapsed transcripts obtained by TAMA are shown. Corresponding results obtained by cDNA cupcake and cogent are shown in Figure A1. Cultivars were sorted alphabetically within the subspecies *aus*, *indica* and *japonica*. IRGSP indicates the Nipponbare reference transcriptome.

**Figure A1:**
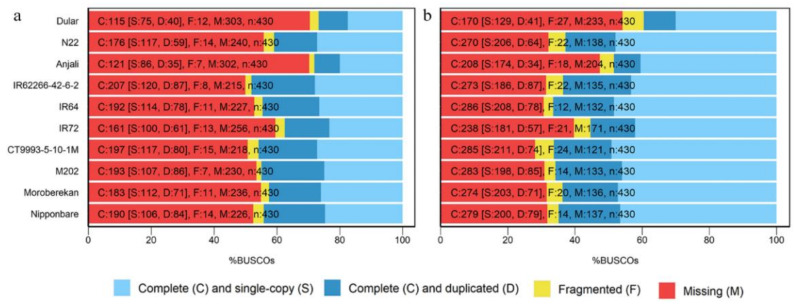
Benchmarking Universal Single-Copy Orthologs (BUSCO) assessment results for the collapsing tools cogent (**a**) and cDNA cupcake (**b**).

Uncollapsed (Figure 1a) and collapsed (Figure 1b) isoforms were evaluated by a BUSCO assessment against a set of 430 highly conserved orthologous proteins in plants and shown for HQ transcripts collapsed with TAMA. Because of the incomplete sampling, between 54% and 27% of the essential proteins were missing, while in the reference transcriptome of Nipponbare (IRGSP) only six essential proteins were missing. The tissue localization of the missing proteins was checked exemplary in the InterPro database [40]. This only provided information on a small fraction of the proteins, but those were mostly expressed in roots, flowers, stems and seedlings, or expressed during a specific developmental stage (Supplementary File S3). Due to our pooling of several RNA samples before library construction, we would also expect to miss rare transcripts due to a dilution effect. For all cultivars, between 3% and 7% of all identified proteins were fragmented before collapsing. This fraction decreased between 2% and 5% after collapsing (Figure 1). Similarly, the number of complete and duplicated transcripts was reduced in favor of single-copy proteins. While for the uncollapsed isoforms, around 19% (Dular) up to 40% (CT9993-5-10-1M) of the proteins were complete and duplicated, this fraction decreased after collapsing to approximately 8% (Anjali) and 18% (IR64) with a corresponding increase of complete and single-copy proteins. For the IRGSP Nipponbare reference transcriptome the majority of transcripts encoded complete and single-copy proteins. Similar results were obtained for cDNA cupcake (Figure A1b). For cogent (Figure A1a) more than 50% of the BUSCO proteins were missing, most likely due to not mapping back to the reconstructed genome.

**Figure 2:**
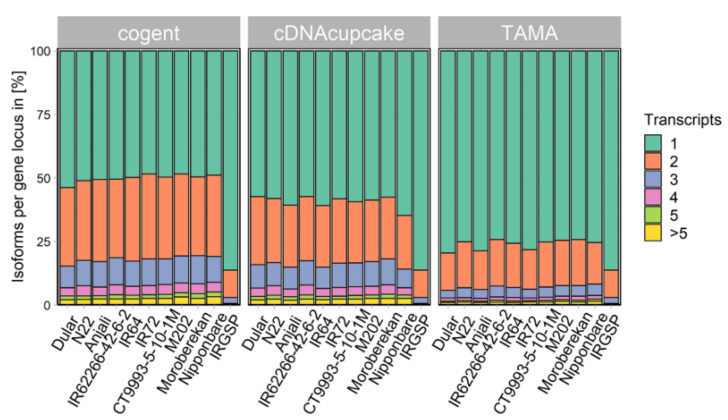
Fraction of isoforms per gene locus for the ten *Oryza sativa* L. (*Poaceae*) cultivars and the Nipponbare reference transcriptome (IRGSP). Cultivars were sorted alphabetically within the subspecies *aus*, *indica* and *japonica*.

**Figure A2:**
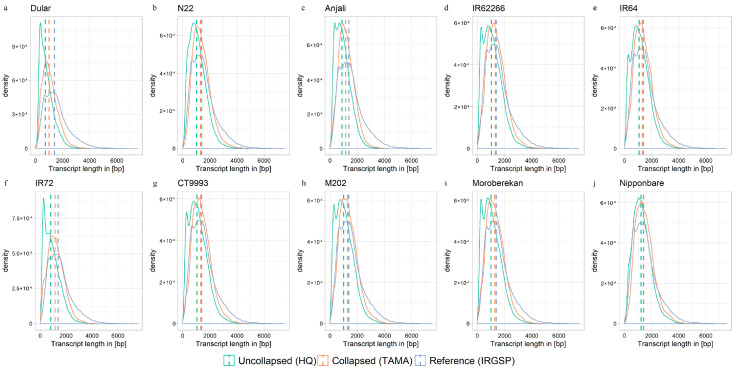
Transcript length distribution for ten *Oryza sativa* L. (*Poaceae*) cultivars. Length distribution of uncollapsed transcripts is indicated in green, length distribution of transcripts after collapsing by TAMA is indicated in orange and length distribution of the Nipponbare IRGSP reference transcriptome is indicated in purple. Dashed lines show the median length of transcripts for the respective datasets.

Through collapsing, the median transcript length increased for all cultivars and for all three methods, as shown for TAMA in Figure A2. The length distribution and median length of the transcripts from each cultivar were more similar to the Nipponbare reference transcriptome after collapsing. Additionally, the number of isoforms per gene locus was determined for all three collapsing methods (Figure 2). TAMA yielded the highest fraction of unique isoform models per gene locus, with around 75% for each cultivar. cDNA cupcake resulted in around 60%, whereas cogent, the reference-free approach, collapsed around 50% of the HQ isoforms into unique isoform models. The relative number of isoforms per gene locus was also determined for the Nipponbare reference transcriptome (IRGSP) resulting in 85% unique isoform models per gene locus.

**Figure 3:**
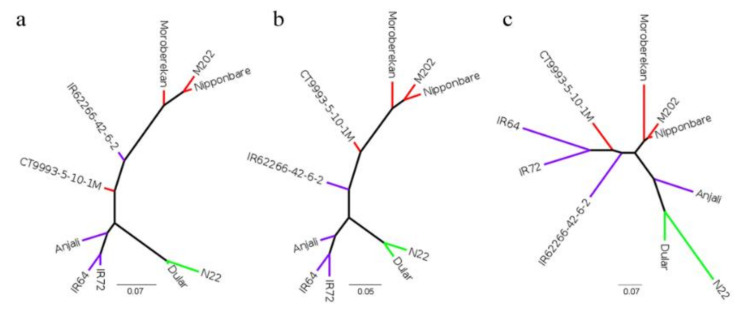
Phylogenetic trees constructed with SNPhylo. Trees are based on SNPs from the transcriptomes of ten *Oryza sativa* L. (*Poaceae*) cultivars from the subspecies *aus*, *indica* and *japonica* after collapsing redundant transcripts with cogent (**a**), cDNA cupcake (**b**) and TAMA (**c**). Red—*japonica*, purple—*indica*, green—*aus*.

The three *O. sativa* subspecies *aus*, *indica* and *japonica* differ in their genome sequences and cultivars from the same subspecies are more closely related [15]. To evaluate genetic distances among our candidate cultivars and to compare the effect of collapsing by different tools, a phylogenetic study was performed. Single nucleotide polymorphisms (SNPs) were called in the collapsed transcriptome datasets based on the IRGSP Nipponbare genome reference and phylogenetic trees were drawn based on an analysis with SNPhylo (Figure 3). SNPhylo extracts high-quality and representative SNPs for the analysis and resulted in around 30,000 SNPs for cDNA cupcake, 23,200 SNPs for cogent and around 16,000 SNPs for TAMA. For all three approaches, the cultivars of the same subspecies clustered together. The trees constructed from the cogent (Figure 3a) and cDNA cupcake (Figure 3b) analyses were more similar to each other than to the tree obtained after collapsing with TAMA (Figure 3c). By all three approaches, the *aus* cultivars were clearly separated from the *indica* and *japonica* cultivars. However, the separation between cultivars of the *indica* and *japonica* subspecies was less clear for cogent and TAMA than for cDNA cupcake.

### 2.3. Evaluation of Reconstructed Transcriptomes

For further biological analysis, collapsed HQ transcripts obtained with TAMA were used. Because TAMA only collapses transcripts mapped against the reference genome, unmapped transcripts were collapsed additionally with cogent. The combined data for each cultivar resulted in 10,511 (Dular) to 15,011 (IR64) reconstructed gene loci as well as between 14,255 (Dular) and 20,803 (Moroberekan) unique isoform models (Table 4). Compared to the Nipponbare transcriptome reference (IRGSP), around one third of the gene loci and about half of the transcript models were reconstructed. The average number of transcripts per gene locus was about 1.4 to 1.5 for each cultivar, which was slightly higher than for the reference transcriptome with 1.2. The median transcript length ranged from 986 bp (Dular) to 1394 bp (Nipponbare) and was similar to the Nipponbare reference of 1385 bp. The average GC content was between 50.87% (Dular) and 52.76% (IR64), again similar to the reference GC content of 51.24%.

**Figure A3:**
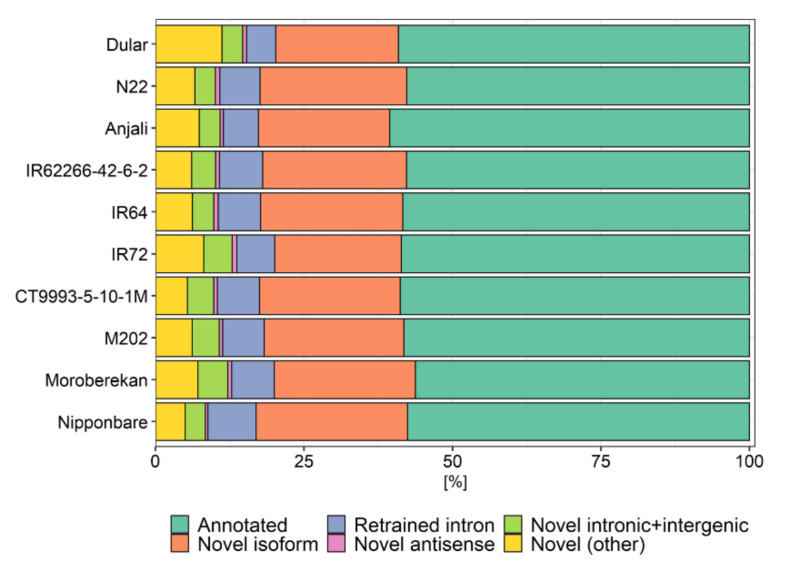
Annotated and novel transcripts as identified by gffcompare using the Nipponbare reference annotation. Cultivars were sorted alphabetically within the subspecies *aus*, *indica* and *japonica*.

The de novo reconstructed transcriptomes of the ten *O. sativa* cultivars were compared with the existing Nipponbare reference annotation using gffcompare. This tool reports transcripts that fully match, partially match or do not match a reference transcript. A full match transcript has an exact intron-exon-chain matching (“Annotated”) to the reference annotation, whereas partially matched transcripts share at least one splice junction with the reference transcript or show intron retention (“Novel isoform”, “Retrained intron”). Additionally, gffcompare also reports isoforms on the antisense strand (“Novel antisense”) compared to the reference, fully contained exon-chains within a reference intron (“Novel intronic”) and on intergenic (“Novel intergenic”) regions as well as intron matches on the opposite strand, exonic overlap on the opposite strand, and others (“Novel other”). About 60% of our reconstructed transcripts were fully matched to a known reference transcript of Nipponbare, while around 40% were reported in a broader sense as novel (Figure A3).

### 2.4. Functional Annotation

**Figure 4:**
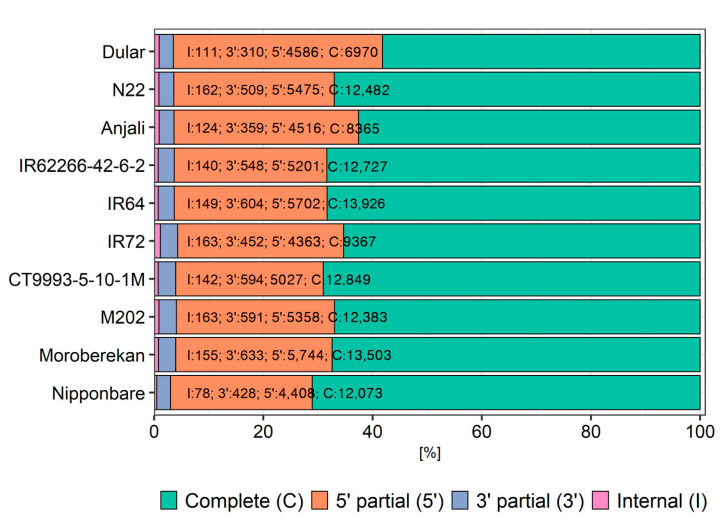
Fraction of predicted open reading frames (ORFs) using TransDecoder. Complete ORFs include start and stop codon, 5′ partial/3′ partial ORFs contain only the start or the stop codon, respectively, and internal ORFs contain neither start nor stop codon. Numbers represent the number of transcripts for each category per cultivar. Cultivars were sorted alphabetically within the subspecies *aus*, *indica* and *japonica*.

To get insight into the biological context of the reconstructed transcripts, functional annotation was performed. Open reading frames (ORFs) were predicted using TransDecoder (Figure 4), including blast and PFAM searches, indicating the presence of approximately 60% to 70% complete ORFs (including start and stop codon). Between 26% and 38% 5′ partial ORFs (only start codon), and low percentages of 3′ partial (only stop codon) and internal (neither start nor stop codon) ORFs were additionally identified.

**Figure 5:**
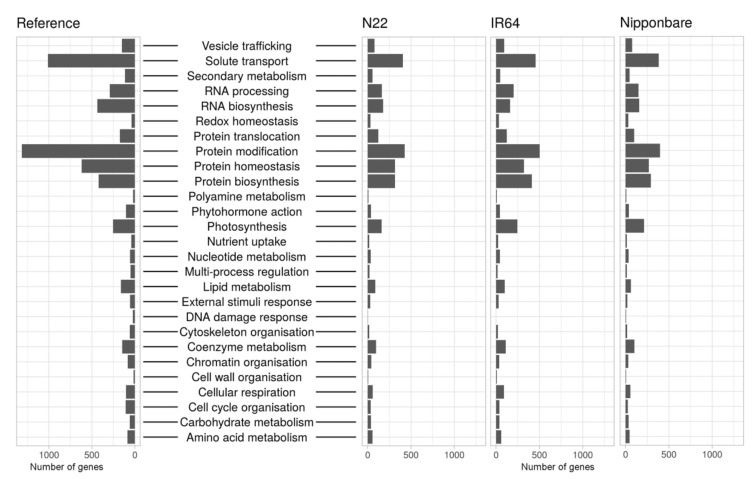
Classification of transcripts into functional bins. Transcripts of N22 (*aus*), IR64 (*indica*) and Nipponbare (*japonica*) were classified into functional bins using Mercator. The bins “not assigned.annotated” and “not assigned.not annotated” are not included.

**Figure A4:**
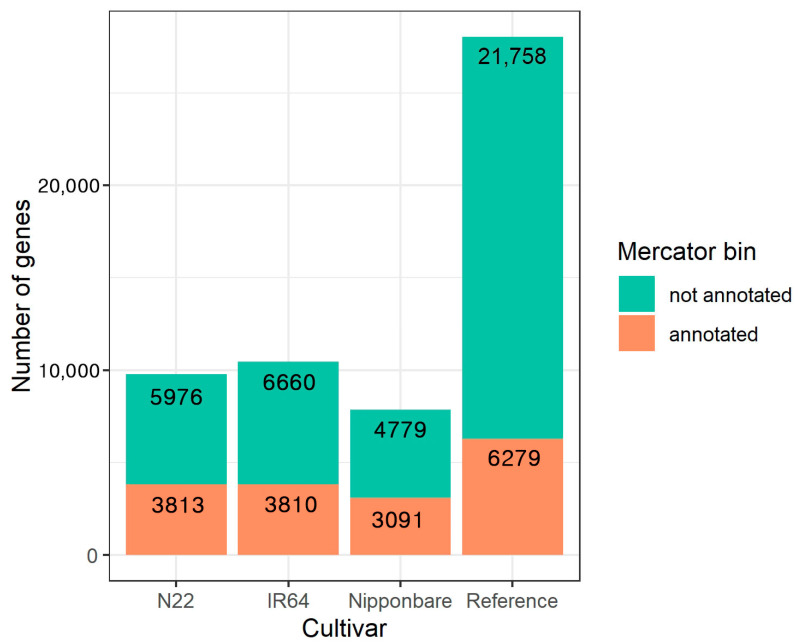
Number of genes classified as “annotated” or “not annotated” among the genes not assigned to a functional bin in the Mercator ontology for rice. Data are shown for the transcriptomes of N22, IR64 and Nipponbare and the Nipponbare reference transcriptome.

Functional annotation was performed with Trinotate and Mercator4. Mercator4 was developed specifically for plants and uses a simple hierarchical tree structure of terms referred to as “bins” that describe biological concepts [41]. Major biological processes such as photosynthesis are represented by top-level bins and each offspring bin describes a more narrowly focused subprocess, ending at the single-protein level for each parent bin. Currently, the ontology comprises 27 functional top-level categories representing a diverse range of biological processes in plants. The number of annotated sequences in each Mercator bin for the cultivars N22, IR64 and Nipponbare as representative cultivars for each subspecies, were compared with all known genes for *O. sativa* in the Mercator ontology (Figure 5). The relative distribution is similar among the three cultivars, and to the reference. However, the Mercator ontology has over 28,000 known *O. sativa* genes (Figure A4) that have not been assigned to a functional bin and hence, between approximately 8000 and 10,000 transcripts were not assigned to functional bins for the three cultivars.

The complete results of the functional annotation using the TransDecoder-Trinotate pipeline and Mercator are shown for each cultivar (available online: 10.6084/m9.figshare.c.5128859). The fraction of sequences with at least one significant hit are summarized in Table 5. For Mercator, blastx, blastp and PFAM retrieved between approximately 60% and 75% significant hits for annotations. For GO terms, only around 38–48% of the transcripts of each cultivar were connected to a functional annotation. Finally, between about 17% and 28% of the transcripts could not be functionally annotated. Because the Swiss-Prot database was used for annotation, which only includes manually curated proteins, data of *Oryza* wild species were mainly not represented. To investigate, whether unannotated transcripts were derived from wild ancestors of *O. sativa*, cDNA sequences of all available *Oryza* wild species were downloaded from EnsemblPlants and compiled as a blast database. Unannotated transcripts were searched against it and between 82% and 92% of these transcripts were highly similar to cDNA sequences of *Oryza* wild species.

### 2.5. Common and Specific Transcripts among Cultivars

**Figure 6:**
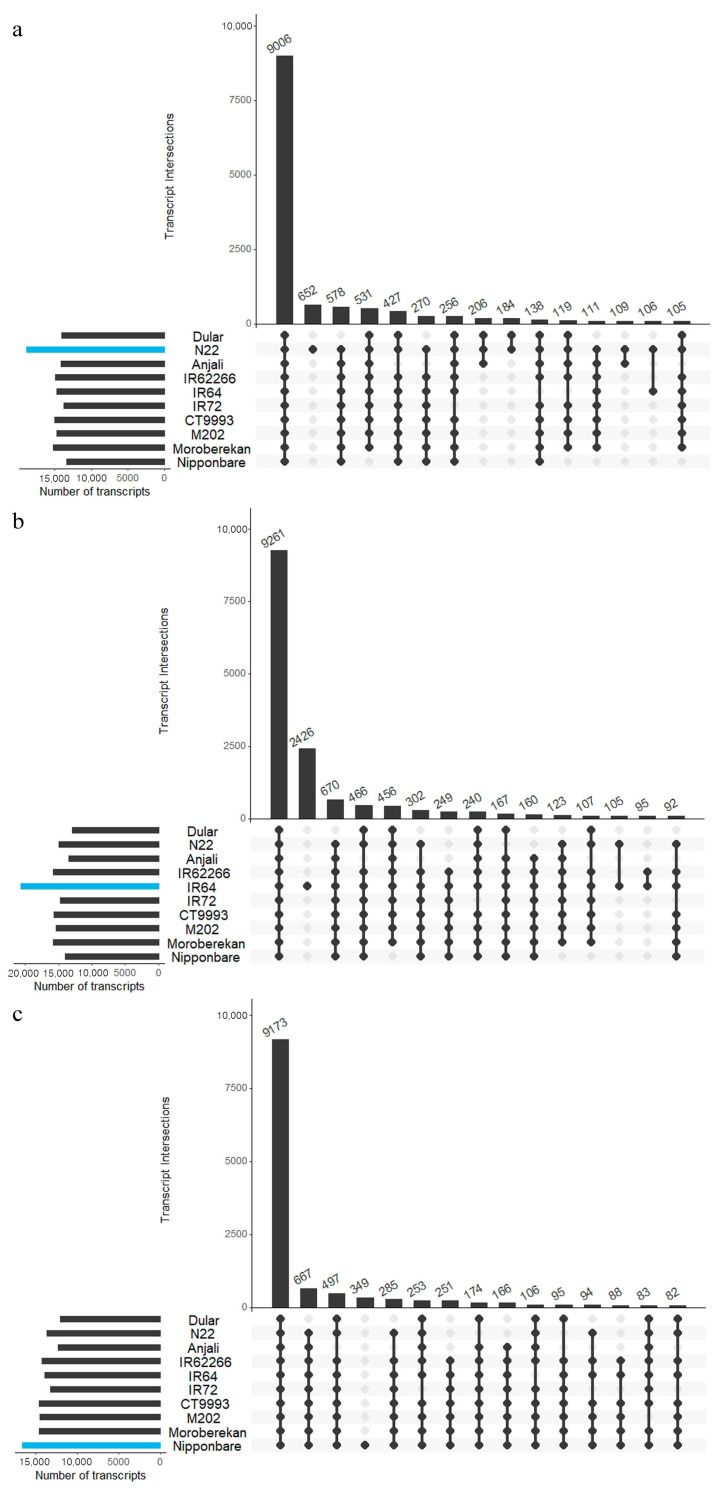
Identified common and specific transcripts over all cultivars. Sequence similarities were identified by a blastn search using the transcriptome of a representative cultivar of each subspecies as database. The best hit for each database entry was selected based on the cultivars N22 (**a**), IR64 (**b**), and Nipponbare (**c**). The 15 largest categories were visualized in an UpSet plot. The barplots on the left of the cultivar names represent the size of the datasets, with the blue bars indicating the size of the search space. Dots and vertical lines indicate the cultivars included in the overlap. Barplots in the top panels represent the number of transcripts in the respective comparison. Cultivars were sorted alphabetically within the subspecies *aus*, *indica* and *japonica*.

To identify cultivar-specific transcripts, the transcriptome of one cultivar of each subspecies (N22, IR64, Nipponbare) was used as a blast database and the sequences of the remaining nine cultivars were searched against it. The most highly significant hit for each database entry of each cultivar was selected and the common overlap with all other cultivars was determined (Figure 6). For N22 (Figure 6a) around 18,000 transcripts were included in the database, of which about 9000 were highly similar to transcripts from the other nine cultivars. In total, 652 transcripts were unique to N22 and over 184 transcripts were only found in the *aus* cultivars N22 and Dular. The *aus*-specific transcripts were extracted, including their annotations (Supplementary File S4). For the *indica* cultivar IR64 (Figure 6b) and the *japonica* cultivar Nipponbare (Figure 6c) the search space included approximately 15,000 and 20,000 transcripts each, resulting also in around 9000 common transcripts over all cultivars. While for IR64 2426 cultivar-specific transcripts were identified, only 349 were determined for Nipponbare (Supplementary File S4).

### 2.6. Differential Gene Expression Analysis for Aus Specific Transcripts

The *aus* cultivar N22 is particularly tolerant to combined drought and heat stress [34]. We therefore asked whether any of the identified *aus*-specific transcripts were regulated under these conditions. A differential gene expression (DGE) analysis was performed for N22 plants grown in the field under control and combined drought and heat stress. RNA-Seq was performed using RNA isolated from developing seeds and the resulting Illumina reads were mapped against the de novo reconstructed N22 transcriptome. After identifying significantly differentially expressed genes with DESeq2 (FDR *p* < 0.1, absolute log_2_ fold-change ≥ 1), 56 *aus*-specific genes were extracted (Supplementary File S5). As determined by a blast search, about 46% of these genes had *Arabidopsis thaliana* (L.) Heynh. (*Brassicaceae*) homologs, 27% lacked any annotation and 11% each were either only described by a PFAM domain or were homologous to sequences in other plant species, while 5% had known homologs in *Oryza*.

**Figure 7:**
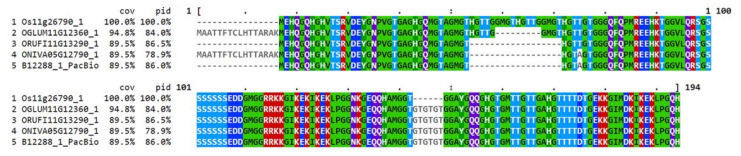
Multiple sequence alignment of five *Oryza* RAB21 dehydrin proteins. Os—*Oryza sativa* L. ssp. *japonica*, OGLUM—*Oryza glumaepatula* Steud., ORUF—*Oryza rufipogon* Griff., ONIVA—*Oryza nivara* S.D. Sharma and Shastry, B12288_1_PacBio—RAB21 protein from the *Oryza sativa* ssp. *aus* cultivar N22. The encoding transcript was identified as *aus*-specific in our analysis. Color theme is “identity” by MVIEW [45].

**Figure A5:**
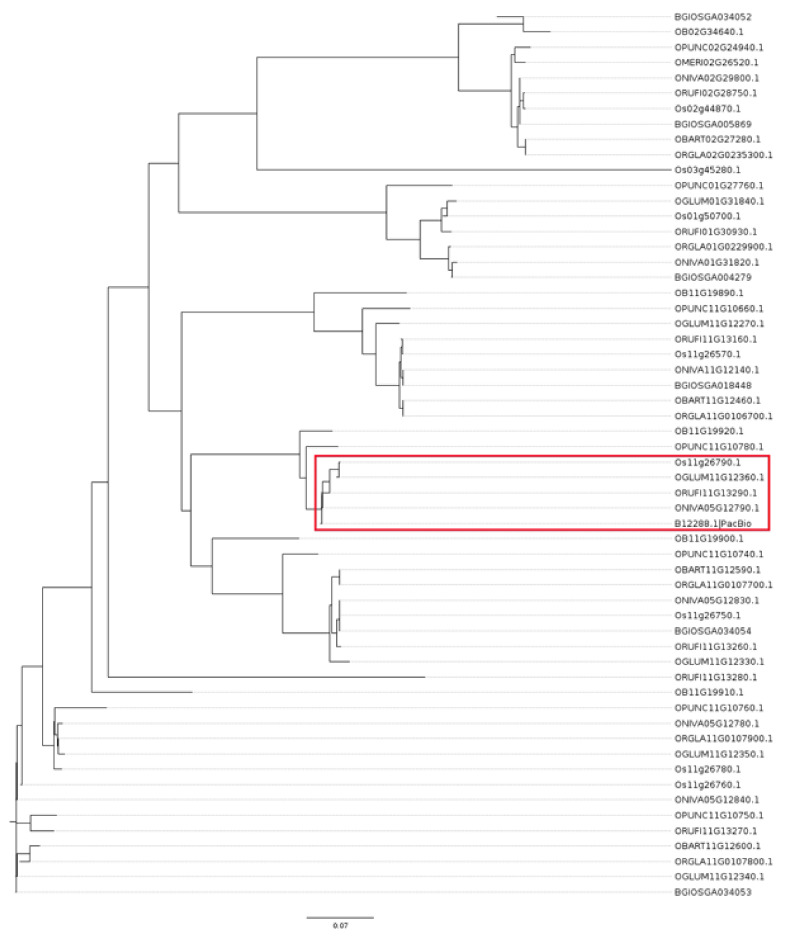
Phylogenetic tree of *Oryza* dehydrin proteins. Dehydrin selection was based on Verma et al. (2017) and includes the following *Oryza* species: BGI—*Oryza sativa* L. ssp. *indica* Kato, Os—*O. sativa* L. ssp. *japonica*, OB—*O. brachyantha* A. Chev and Roehrich, OPUNC—*O. punctate* Kotschy ex Steud., OGLUM—*O. glumaepatula* Steud., ORUF—*O. rufipogon* Giff., ORGLA—*O. glaberrima* Steud., ONIVA—*O. nivara* S.D. Sharma and Shastry, OBART—*O. barthii* A. Chev, OMERI—*O. meridionalis* Ng. The red box indicates the proteins used for the sequence alignment shown in Figure 7.

As an example, we describe the gene *B12288*, which was significantly upregulated during combined heat and drought stress (Supplementary File S5). It has homologous genes in both *japonica* and *indica* cultivars annotated as *RAB21*. The gene is induced by drought and the corresponding protein belongs to the dehydrin family of Late Embryogenesis Abundant (LEA) proteins. Evolutionary relationships with other *Oryza* dehydrins [42] were investigated by multiple sequence alignments and visualized as a tree (Figure A5). The N22 gene product was closely related to four other dehydrins in wild rice species and *O. sativa* ssp. *japonica*. It showed 89.5% sequence coverage and 86.0% sequence identity compared to the *japonica* protein (Figure 7) including the highly conserved repeat regions characteristic of dehydrins [43,44]. The N22 protein was more similar to the proteins from *Oryza* wild species than to the *japonica* protein (see Figure 7 and Figure A5).

## 3. Discussion

### 3.1. Sequencing Performance

Between 15.7 and 24.5 GB of cDNA were sequenced for each cultivar on two or three SMRT cells resulting in from 460,340 up to 736,747 full-length non-chimeric (FLNC) reads. Using the IsoSeq3 protocol, between 38,000 and 54,700 high-quality (HQ) transcripts for each cultivar were obtained before filtering out contaminants. Among the ten cultivars, the sequencing output was similar. In previous studies of plant transcriptomes more SMRT cells were used, but this resulted in a similar output of FLNC reads and HQ transcripts. For example, for the wheat cultivar Xiaoyan 81 [46] around 197,800 FLNC reads were obtained on the RSII platform and processed into 91,800 HQ reads based on eight SMRT cells. With the newer PacBio Sequel platform that we also used in our study, around 650,000 FLNC reads were obtained using five SMRT cells analyzing the transcriptome of the wild cotton species *Gossypium australe* F. Müll. [29] but in this case an older chemistry and software were used. Therefore, it is difficult to directly compare the sequencing output from different studies. However, our results indicate that two to three SMRT cells are sufficient to obtain useful Iso-Seq data with the currently available technology.

The PacBio technology has a relatively high sequencing error-rate, but these errors are distributed randomly among the sequence [24]. Since sequencing is performed on circularized cDNA molecules, several sequencing passes can be generated for a given cDNA, carrying errors in different random locations. The PacBio IsoSeq3 tool is then generating a consensus sequence based on the multiple sequenced cDNA template to eliminate these errors. However, even after the correction, InDels and SNPs may still occur. In a study on sorghum [25] using the older RSII technology, HQ reads were mapped against a reference genome sequence and a per-nucleotide error rate of 2.34% was observed. This made a correction using corresponding RNA-Seq data necessary. Using the Sequel technology, we found a per-nucleotide error rate between 0.08% and 0.14% for the uncorrected HQ reads, based on mapping against the respective subspecies reference genome sequences. This low error rate made further correction unnecessary.

### 3.2. Collapsing Redundant Transcripts and Transcriptome Quality Assessment

During library preparation, degradation products can be formed and are subsequently sequenced. These shorter transcripts lack some of the 5′ sequence but are otherwise identical to the full-length transcripts, resulting in large numbers of redundant transcripts. This effect can be reduced experimentally using specific 5′ end capturing library preparation methods, or it can be partly compensated computationally by the use of collapsing software. We compared the utility of the tools cogent, cDNA cupcake and TAMA to reduce the number of redundant transcripts. Cogent does not need a reference genome sequence to collapse redundant isoforms and was successfully applied to transcriptomes from organisms without an available genome reference [29,47,48]. cDNA cupcake and TAMA, on the other hand, need a reference genome sequence and have been more commonly used [49,50,51,52].

In our study, the number of transcripts after collapsing decreased by up to 68%, indicating the necessity to reduce redundancy and thereby improve data quality. While TAMA and cogent resulted in similar numbers of collapsed transcripts, the numbers were slightly higher after processing with cDNA cupcake. Cogent left more transcripts unmapped, compared to the other tools. This may be due to the generation of transcript orphans, i.e., putative single-isoform transcripts that were not incorporated into the reconstructed transcriptomes.

Transcriptome quality improvement after collapsing was shown by the BUSCO assessment, where the number of encoded complete and single-copy proteins increased by approximately 20% to between about 35% and 55% of all proteins included in BUSCO, while for the reference transcriptome this was about 75%. However, as expected, only about 70% of all BUSCO proteins were covered by our partial transcriptomes. For comparison, PacBio sequencing of the sugarcane transcriptome [28] using a pooled RNA sample derived from leaf, internode and root tissues at different developmental stages collected from 22 varieties resulted in a coverage of 90% of the BUSCO proteins. However, since no collapsing was performed, this study found 66% complete but duplicated BUSCO proteins.

Collapsing transcripts with TAMA resulted in the highest fraction of one isoform models per gene locus and the average number of isoforms per locus in our different transcriptomes was very similar to the Nipponbare reference transcriptome. This is, however, not always the case. A PacBio IsoSeq study in maize [53] identified an average of 6.56 isoforms per gene locus using the cDNA cupcake pipeline, more than twice the number found for the reference genome annotation with an average of 2.84 transcripts per gene locus. Cogent and cDNA cupcake yielded lower fractions of one isoform models per gene locus in our study. Since there are, to the best of our knowledge, no other direct comparisons of the three collapsing tools available, it cannot be judged whether the tools may perform differently with different data sets or different reference transcriptomes. Obviously, only cogent could be used in cases where no reference genome sequence is available.

Around 70% of the transcripts covered a complete ORF in most of the cultivars. Only Dular and Anjali showed a smaller fraction of complete ORFs. The differences among the cultivars are due to a different fraction of 5′ truncated ORFs. In these cases, either the collapsing tool (TAMA) has not worked sufficiently, or no full-length ORFs were sequenced for these particular transcripts. Either way, it seems that a certain fraction of incomplete ORFs cannot be avoided, given the methodology we employed in our study. A PacBio IsoSeq study of the chicken transcriptome compared brain and embryo RNA libraries, where both libraries were normalized to reduce over-represented transcripts, but only for the embryo library a 5′ cap selection was performed [54]. Here, the number of transcripts dropped by 60% for the brain data and by 21% for the embryo data after collapsing with an older version of cDNA cupcake, indicating lower transcript redundancy for the capped library. However, it remains to be tested in detail, whether other library preparation methods would yield better results, perhaps in combination with the collapsing approach.

### 3.3. Common Transcripts and Differential Gene Expression Analysis

Even for the well-annotated Nipponbare transcriptome, around 17% of the transcripts that we found did not have a functional description and are therefore considered to be novel isoforms. Similarly, for the remaining cultivars, between 19% and 28% of the transcripts could not be assigned with a functional description. This is supported by the identification of a large fraction of potential novel isoform models by the gffcompare tool compared with the Nipponbare reference transcriptome. However, gffcompare also reports isoforms as “novel” models, which share at least one splice junction with the reference transcript and differ in the remaining splice junctions for multiple-exon transcripts. This criterion can be weak for example where exon-exon boundaries are shifted due to sequencing errors [55].

Since all ten cultivars that we analyzed belong to the same species, they should have a large fraction of common transcripts that may be identified by a blast search. We therefore used the transcriptome of one cultivar from each subspecies to generate a database for blast searches of the other nine transcriptomes. With this approach, we were able to identify common, cultivar- and subspecies-specific transcripts within our datasets. It must be stressed, however, that the lack of a transcript in the transcriptome of a particular cultivar may have two reasons. It could indeed be absent from the transcriptome and genome of this cultivar, or it could be missing from the transcriptome of this cultivar relative to one of the databases because of differences in sampling, such as different tissues or growth conditions.

Our analysis indicated, as expected, that the largest fraction of the transcripts identified in N22 (47.6%), IR64 (44.8%) and Nipponbare (55.2%) were common to all transcriptomes. Using the *aus* cultivar N22 as the database yielded 652 N22-specific and an additional of 184 *aus* specific transcripts, resulting in 836 transcripts in total (4.4% of the total N22 transcripts) that were only found in the *aus* cultivars. Interestingly, we also identified 160 transcripts in IR64 and 166 in Nipponbare that were not present in either of the *aus* transcriptomes, while neither the IR64 nor the Nipponbare transcriptomes contained any transcripts that were specific for the respective subspecies. The Nipponbare transcriptome only contained a very small fraction (2.1%) of cultivar-specific transcripts. This was very different in the IR64 transcriptome with over 2426 unique transcripts, comprising 11.7% of the transcriptome. We attribute this high fraction of cultivar-specific IR64 transcripts to the fact that only in this case roots were included in the analysis and submergence and salt stress were applied. In all other cultivars, only above-ground tissues were sampled, and treatments involved exclusively high night temperatures, heat and drought stress.

*Aus* cultivars are known to be more stress tolerant than *indica* or *japonica* cultivars and contain genes, such as the phosphate starvation tolerance gene *OsPSTOL1* [17], the submergence tolerance gene *OsSUB1A* [18] and the deepwater escape genes *OsSNORKEL1/2* [19] that are absent in the Nipponbare reference genome. To test whether our transcriptome sequencing approach might aid in the identification of such *aus*-specific stress-related genes, we performed a differential gene expression analysis by Illumina-based RNA-Seq. The samples from developing seeds were obtained from N22 plants grown under control and combined drought and heat stress in the field [34]. More than 50 significantly differentially expressed genes were identified as unique to the *aus* subspecies transcriptomes. Over 45% of the gene products were annotated as homologous to an *A. thaliana* gene, such as the gene *B12989* annotated as encoding a RALF precursor polypeptide, which may regulate plant stress responses, growth and development in Arabidopsis and tobacco (*Nicotiana tabacum* L.) [56].

We characterized one of the significantly induced genes in more detail. The gene *B12288* is annotated as *RAB21*. This gene has homologs in different *O. sativa* subspecies and in various wild species of *Oryza*. It belongs to the dehydrin family of LEA proteins and high levels of expression of *RAB21* have been found in mature seeds, as well as in vegetative tissues under salt and drought stress, and after treatment of rice seedlings with the plant stress hormone abscisic acid [57]. The drought and heat induced *RAB21* gene we identified in N22 was more closely related to *RAB21* isoforms from wild rice species than to the homolog from Nipponbare. The sequence differences are not large but may nevertheless be functionally significant. It has been shown with in-vitro assays that some dehydrins are able to protect enzymes from inactivation under heat stress [58,59], indicating a possible function of RAB21 under combined drought and heat stress conditions that led to transcriptional upregulation. It is still unclear which structural characteristics determine the ability of a dehydrin to act as an enzyme stabilizer under heat stress and therefore, the functional significance of the sequence differences between RAB21 from Nipponbare and N22 cannot be evaluated. However, it has recently been shown that changes in only four amino acids in the LEA protein COR15A from Arabidopsis significantly increased the stabilizing effect of this protein for membranes during freezing [60]. It is therefore conceivable that the minor differences in amino acid sequence between the RAB21 proteins from different subspecies and wild rice species may have significant functional effects. Obviously, further experimental work will be necessary to test this hypothesis.

## 4. Materials and Methods

### 4.1. Plant Material

Different tissues of ten cultivars from the *O. sativa* ssp. *japonica*, *indica* and *aus* were used for RNA isolation. Cultivars of the subspecies *aus*, *indica* and *japonica* are referred to in the text as *aus*, *indica* or *japonica* cultivars. Plants were grown under combined drought and heat stress in the field at the International Rice Research Institute at the Philippines (IRRI) (Dular, N22, Anjali) [34], under heat and combined drought and heat stress under controlled climate conditions at IRRI (N22, Moroberekan) [35], under shoot submergence and root salinity, and combined shoot submergence and root salinity in net-houses at IRRI (IR64) [32], under high night temperature stress under controlled climate conditions at the Max Planck Institute of Molecular Plant Physiology in Germany (IR62266-42-6-2, IR64, IR72, CT9993-5-10-1M, M202, Moroberekan, Nipponbare) [36] and under high night temperature stress in the field at IRRI (IR62266-42-6-2, IR64, IR72, CT9993-5-10-1M, M202, Moroberekan) [33]. Samples were obtained from plants grown under both stress and control conditions (see Supplementary File S1 for a complete list of all samples). An overview of cultivars, tissues and growth environments is given in Table 1. The selection of cultivars was based on their different sensitivity to high night temperature [36], heat, drought or combined heat and drought stress [34,35].

### 4.2. RNA Extraction and Sequencing

Total RNA was isolated from homogenized frozen material from all samples listed in Supplementary File S1 using Trizol-based methods [61,62]. RNA was quantified spectrophotometrically (NanoDrop Technologies, Wilmington, DE, USA) and genomic DNA contamination was removed by DNase treatment (Rapid Out DNA Removal Kit, Thermo Scientific, Dreieich, Germany). Absence of genomic DNA was verified by qRT-PCR using a primer pair amplifying an intron sequence [63]. Final RNA quality and integrity were assayed using the Agilent 2100 Bioanalyzer (Agilent Technologies, Santa Clara, CA, USA). For each cultivar, RNA isolated from all organs and treatments was pooled to generate one sample per cultivar. PacBio library preparation and sequencing were performed at the Max Planck Genome Center Cologne, Germany. cDNA was synthesized and amplified according to the Pacific Biosciences’s protocol using the SMARTer PCR cDNA Synthesis kit (Clontech, Mountain View, CA, USA) and amplification by the KAPA HIFI PCR Kit (Kapa Biosystems, Wilmington, MA, USA). The cDNAs were not size-selected and PacBio libraries were prepared with the SMRTbell Template Prep Kit 1.0 (Pacific Biosciences, Menlo Park, CA, USA) and sequenced on the PacBio Sequel I with Sequel DNA polymerase and binding kit and sequencing chemistry version 2.1 for 600 min. Each library was sequenced on two or three SMRT cells to achieve sufficient coverage.

For RNA-Seq analysis, RNA was isolated from developing seeds of the *aus* cultivar N22. Plants were grown in the field in 2013 under either well-watered control conditions or under combined drought and heat stress [34] and RNA was extracted using Ribospin Seed/Fruit and Riboclear *plus!* (GeneAll Biotechnology, Songpa-gu, Republic of Korea) following the manufacturer´s instructions. Three biological replicates were generated for each condition (control/stress). Quantification of RNA and quality controls were performed as described above. Library preparation and sequencing were performed at the Max Planck Genome Centre Cologne. Libraries were prepared with NEBNext Ultra Directional RNA Library Prep Kit for Illumina (New England Biolabs, Frankfurt am Main, Germany) and sequenced using Illumina HiSeq 3000 technology generating approximately 30 million 150 base pair-long single-end reads per sample.

### 4.3. De novo Transcriptome Reconstruction

To generate full-length isoforms, the software IsoSeq3 v3.0 included in smrtlink v5.1 (Pacific Bioscience, Menlo Park, CA, USA) was used to perform the following four steps: consensus (ccs 3.0.0), lima (lima 1.0.0), cluster (sierra 0.7.1) and polish (tango 0.7.1). Raw data processing for each library was performed on combined data from two or three SMRT cells (using the smrtlink command create) with default parameters:

ccs $in.subreads.bam $out.bam --noPolish --minPasses=1
lima $in.xml primer.fasta $out.demux.ccs.bam --isoseq --no-pbi --dump-clips
isoseq3 cluster $in.demux.ccs.bam $out.unpolished.bam
isoseq3 polish $in.unpolished.bam $out.polished.bam

As final output high-quality (HQ) and low-quality (LQ) isoforms were obtained. Only HQ isoforms were used for subsequent analysis. To identify contaminations, HQ isoforms of all cultivars were aligned against the NCBI nucleotide database (downloaded: 24.07.2018) with blastn v2.3.0 [38] (E ≤ 1 × 10^10^). Isoforms without a hit were aligned against the NCBI protein database (downloaded: 24.07.2018) using blastx v2.3.0 [38] (E ≤ 1 × 10^−10^). All isoforms without a significant hit for the family *Viridiplantae* (green plants) were defined as contaminations and removed.

### 4.4. Genome References

For insertion and deletion (InDel) determination, collapsing and mapping, three *O. sativa* genome references from the subspecies *aus* (N22) [6], *indica* (Shuhui498 (R498 genome)) [13] and *japonica* (Nipponbare, IRGSPv1.0.44) [10] were used.

### 4.5. InDel Analysis

HQ isoforms of each cultivar were mapped against the subspecies-specific reference genomes using minimap2, v2.17-r941 [64] with the parameters --ax splice, --uf --C5 and --secondary=no. Insertions and deletions were determined by extracting the cigar string from the alignment files in bam format [65].

### 4.6. Collapsing Redundant Isoforms

For the removal of redundant PacBio isoforms, three tools were tested, namely Transcriptome Annotation by Modular Algorithms (TAMA) [50], cDNA cupcake [66] and COding GENome reconstruction Tool (cogent v3.9) [67] followed by the cDNA cupcake collapse pipeline. For further descriptions, we will refer to the latter only as cogent. TAMA and cDNA cupcake use a reference genome to collapse PacBio isoforms, while cogent employs a reference-free approach, where it reconstructs gene loci based on PacBio isoforms creating its own “coding genome”. Afterwards, cDNA cupcake is employed to collapse the isoforms based on the created reference. For TAMA, the following parameters were used: -x no_cap,-e longest_ends,-a 100,-z 100,-m 30 and -d merge_dup. cDNA cupcake and cogent were run with default parameters following the descriptions on the corresponding websites [68,69]. For both reference-based approaches, the respective reference genome of the appropriate subspecies was used, and HQ isoforms were mapped with minimap2 v2.17-r941 [64]. For all downstream analysis, collapsed transcript models obtained by TAMA were used. While cogent and cDNA cupcake provide the PacBio transcripts after collapsing, TAMA generates a bed file with the coordinates of the collapsed transcripts and sequences extracted from the corresponding genome sequence of each subspecies for the ten cultivars using bedtools v2.27.0 [70] getfasta. Additionally, remaining unmapped transcripts were collapsed with cogent and added to the final datasets. All collapsed datasets are available online [71].

### 4.7. BUSCO Analysis

A set of 430 *Viridiplantae* conserved ortholog proteins was used in BUSCO v3.0.2 (Benchmarking Universal Single-Copy Orthologs) [72] to assess the completeness of the conserved content of the de novo reconstructed transcriptomes using the BUSCO transcriptome mode.

### 4.8. Phylogenetic Analysis

For phylogenetic analysis, SNPs of the collapsed transcripts from TAMA, cDNA cupcake and cogent were used for analyses with SNPhylo [73]. Collapsed transcripts of all cultivars obtained by cogent and cDNA cupcake were mapped against the IRGSP Nipponbare reference genome and SNPs were called utilizing the bcftools v1.9 pipeline [74]. For TAMA, HQ transcripts of all cultivars were collapsed based on the Nipponbare reference genome and the generated variant file was used to determine SNPs. Entries were filtered for the “M” type and defined as alternative alleles. The respective reference alleles were extracted with bedtools v2.27.0 from the reference genome. A simple SNP file was generated and used as input for SNPhylo. Phylogenetic trees were visualized with Figtree [75].

### 4.9. Comparison of Reconstructed Transcriptomes

HQ collapsed sequences were classified and compared with the existing IRGSP Nipponbare annotation using gffcompare v0.11.2 [76]. The classifications defined by gffcompare were generalized into annotated (classes “=” and “c”), novel isoform (classes “j” and “k”), retrained intron (classes “m” + “n”), novel antisense (class “x”), novel intronic/intergenic (classes “i” and “u”) and novel others (classes “o”, “y”, “e”, “s” and “p”).

### 4.10. Functional Annotation

ORFs were predicted with TransDecoder v5.5.0 [77]. The candidate protein coding regions were extracted by transDecoder.LongOrfs with a minimum length of 100 amino acids. Resulting ORFs were characterized according to similarities to known proteins by a blastp v2.3.0 search [38] (E ≤ 1 × 10^−5^) of the comprehensive Swiss-Prot protein database [78] (downloaded 09.09.2019) and for conserved protein domains using Hmmer v3.2.1 [79] based on the Pfam database [80] (downloaded 18.09.2019). Finally, likely coding regions were reported by the transDecoder.Predict module including all peptides with blast or domain hits. Additionally, HQ collapsed transcripts of all ten cultivars were searched against the Swiss-Prot database using blastx v2.3.0 (E ≤ 1 × 10^−10^). All results (blastp, blastx and Pfam) were parsed by Trinotate v3.2.0 [81], stored in an SQLite relational database and then reported as a tab-delimited transcript annotation summary file. Additional Gene Ontology (GO) information was extracted by Trinotate based on the Swiss-Prot database entries. Mercator v4.2 [41] was used as an additional functional annotation pipeline. HQ collapsed nucleotide sequences were submitted online [82] and resulting tables were downloaded. Trinotate and Mercator tables were merged to one table per cultivar and is available online [71]). For a detailed comparison with existing *O. sativa* bins, results were also compared to the rice MSU7 annotation on the Mercator website and saved. All transcripts without any annotation for Mercator or the TransDecoder-Trinotate pipeline were extracted and a blastn search (min. identity 85%, E ≤ 1 × 10^−10^) performed against all available cDNA files of *Oryza* wild species obtained from EnsemblPlants [83].

### 4.11. Determination of Common Overlap

Common overlap of transcripts among the cultivars was determined using blastn v2.3.0 [38] with stricter thresholds than before (E ≤ 1 × 10^−10^; min. identity 95%). The transcriptome data of the cultivars N22, IR64, and Nipponbare were transformed into blast databases and the transcripts of the remaining nine cultivars were searched against these databases. Results were filtered for the best hit for each database entry, and the common overlap was determined and visualized using the R package UpSetR [84].

### 4.12. Differential Gene Expression Analysis

RNA-Seq data for the *aus* cultivar N22 were mapped against the reconstructed PacBio N22 transcriptome using kallisto v0.45 [85]. Based on the mappings, a differential gene expression analysis was performed using the R-package DESeq2 v1.26.0 [86]. *Aus*-specific differentially expressed transcripts were extracted, and transcript annotations merged on gene level. A selected candidate gene (*B12288*) was investigated in more detail. Based on the annotation, the product of *B12288* is a dehydrin and hence, a multiple sequence alignment was performed with rice specific dehydrin sequences [42] using Clustal Omega [45]. The resulting phylogenetic tree was visualized using Figtree [75]. Protein sequences were downloaded from www.uniprot.org. The multiple sequence alignment of four closely related protein sequences to the candidate protein B12288 was visualized with MView [45].

### 4.13. Graphical Visualization

If not mentioned otherwise, the R packages ggplot2 [87], ggpubr [88], gridExtra [89] and reshape2 [90] were used for graphical visualization of the results.

### 4.14. Availability of Data and Material

PacBio raw data are available in the NCBI’s SRA database under the accession number PRJNA640670. Collapsed and filtered HQ sequences and functional annotation of all ten cultivars are available online [71]. RNA-Seq data are available at GEO [91] under the accession number GSE153030.

## 5. Conclusions

The central question of our study was whether targeted partial transcriptomes obtained by PacBio Iso-Seq may be useful for the down-stream RNA-Seq analysis in rice cultivars from subspecies such as *aus*, which are not well represented by the Nipponbare reference genome sequence. Moreover, by using these transcriptomes, we wanted to discover novel transcripts and genes involved in abiotic stress responses in rice. Our analysis has shown that for all cultivars, cultivar-specific transcripts could be identified. In addition, a number of *aus* subspecies-specific transcripts were determined. These results strongly suggest that this approach will be useful for future analysis of RNA-Seq datasets. The general approach should also be suitable for many other plant species for which no high-quality genome assemblies are available, as it represents a much cheaper and computationally less challenging alternative when the aim is the targeted analysis of RNA-Seq data. In principle, the approach should also be applicable to species outside of the plant kingdom. Additionally, interesting candidate genes have been identified (e.g., for N22). These results can be used as a resource to improve the environmental stress tolerance of rice in an effort to generate climate change resilient cultivars through targeted molecular breeding. The transcriptomes that we have reconstructed here will be directly available for the research community.

## Figures and Tables

**Table 1:** Sampling for PacBio isoform sequencing. RNA of ten *Oryza sativa* L. (*Poaceae*) cultivars from different organs and conditions was extracted and pooled for each cultivar (FL—flag leaves, LE—leaves, PA—panicles, FS—flowering spikelets, DS—developing seeds, SH—sheaths, RO—roots, SO-shoots, PP—pollinated pistils, AN—anthers). Seed database accession numbers (IRTP/IRGC/IRIS ID No.) from the International Rice Research Institute (IRRI) are shown. Plants were grown in climate chambers (CC), net-houses (NH), and/or in the field (F). Cultivars were sorted alphabetically within the subspecies (ssp.) *aus*, *indica*, and *japonica*. See Supplementary File S1 for a more detailed description of all samples used for RNA isolation. X—sample was taken for the respective organ and cultivar.

Cultivar	ssp.	ID No.	Organ										Set-up		
			FL	LE	PA	FS	DS	SH	RO	SO	PP	AN	CC	F	NH
Dular	* aus *	IRGC 636	X			X	X							X	
N22		IRTP 3911	X			X	X							X	
Anjali	* indica *	IRTP 23206	X			X	X							X	
IR62266-42-6-2		IRGC 117597	X	X	X	X		X					X	X	
IR64		IRTP 12158	X	X	X				X	X			X	X	X
IR72		IRTP 14747	X	X	X	X		X					X	X	
CT9993-5-10-1M	* japonica *	IRIS 71-1229921	X	X	X	X		X					X	X	
M202		IRGC 77142	X	X	X	X		X					X	X	
Moroberekan		IRGC 12048	X	X	X	X					X	X	X	X	
Nipponbare		IRGC 12731	X	X	X								X	X	

**Table 2:** Overview of results from PacBio full-length isoform sequencing from ten *Oryza sativa* L. (*Poaceae*) cultivars. Identified high (HQ) and low quality (LQ) isoforms were analyzed for non-plant contamination using blast. Contaminating sequences (not in the group of *Viridiplantae*) were removed (HQ after filtering). PB—number of PacBio SMRT cells, GB—total number of sequenced base pairs in gigabases, FLNC—full-length non-chimeric reads. Cultivars were sorted alphabetically within the subspecies (ssp.) *aus*, *indica* and *japonica*.

Cultivar	ssp.	PB	GB	FLNC	HQ	LQ	HQ after Filtering
Dular	*aus*	2	18.46	460,340	42,252	1960	41,396
N22		3	24.17	736,747	54,572	1807	52,333
Anjali	*indica*	2	15.49	481,094	40,208	1732	39,438
IR62266-42-6-2		2	22.48	649,085	50,569	1659	50,510
IR64		2	21.97	622,881	49,633	1279	49,327
IR72		2	20.31	554,872	44,176	2170	44,049
CT9993-5-10-1M	*japonica*	2	20.81	620,595	48,537	1465	48,401
M202		2	24.07	656,740	48,836	1501	48,676
Moroberekan		2	24.51	675,251	54,684	1721	54,594
Nipponbare		3	15.65	544,792	37,951	1233	37,535

**Table 3:** Number of isoform models after collapsing with TAMA, cDNA cupcake and cogent. #Tr.—number of filtered, high-quality isoforms used for collapsing. Cultivars were sorted alphabetically within the subspecies (ssp.) *aus*, *indica*, and *japonica*.

Cultivar	ssp.	Reference	# Tr.	Reference-Based			Reference-Free	
				TAMA	cDNA Cupcake	Unmapped	Cogent	Unmapped
Dular	*aus*	n22	41,396	13,995	18,239	313	13,107	7340
N22			52,333	18,787	23,954	149	19,026	6603
Anjali	*indica*	S498	39,438	14,371	18,170	178	13,237	6476
IR62266-42-6-2			50,510	18,926	23,803	220	18,773	6913
IR64			49,327	19,064	23,435	1911	17,874	7979
IR72			44,049	15,954	20,646	143	15,251	7426
CT9993-5-10-1M	*japonica*	Nipponbare	48,401	18,789	23,415	223	18,359	6611
M202			48,676	18,925	23,670	240	18,091	6695
Moroberekan			54,594	20,604	26,009	268	20,378	7358
Nipponbare			37,535	16,584	19,674	42	14,345	5441

**Table 4:** Summary of reconstructed transcriptomes including the Nipponbare reference transcriptome (IRGSP). #GL—Number of gene loci, #TR—Number of transcripts, #TR/GL—average number of transcripts per gene locus, Total #bp—total number of bp of all transcripts, Min—shortest transcript in bp, Max—longest transcript in bp, Median—median length of transcripts in bp, GC—content of the nucleotides G and C in %. Cultivars were sorted alphabetically within the subspecies (ssp.) *aus*, *indica*, and *japonica*.

Cultivar	ssp.	# GL	# TR	# TR/GL	Total # bp	Min [bp]	Max [bp]	Median [bp]	GC [%]
Dular	*aus*	10,511	14,255	1.4	15,447,641	56	4551	986	50.87
N22		13,343	18,913	1.4	26,290,969	62	5911	1295	52.26
Anjali	*indica*	10,616	14,499	1.4	17,717,403	75	4216	1156	51.99
IR62266-42-6-2		13,227	19,093	1.4	26,791,848	51	7190	1314	51.37
IR64		15,011	20,672	1.4	28,663,408	56	6919	1299	52.76
IR72		11,647	16,081	1.4	19,678,018	53	5475	1149	51.16
CT9993-5-10-1M	*japonica*	13,354	18,963	1.4	26,757,988	55	5752	1318	51.97
M202		13,143	19,105	1.5	26,258,012	59	6644	1287	51.74
Moroberekan		14,324	20,803	1.5	28,446,682	57	7072	1278	51.80
Nipponbare		11,366	16,622	1.5	24,760,098	75	6035	1394	52.60
IRGSP	*japonica*	38,866	45,660	1.2	69,184,066	30	16,029	1385	51.24

**Table 5:** Fraction of transcripts (%) for which at least one significant annotation was found by Mercator or the TransDecoder-Trinotate pipeline (blastx, blastp, PFAM or GO). Furthermore, also shown is the percentage of transcripts for which no annotation was reported. All unannotated transcripts (No annotation) were additionally compared with an *Oryza* wild species cDNA database using blast. The fraction of unannotated transcripts with a highly similar sequence to an *Oryza* wild species cDNA is shown (Homologs WS). Cultivars were sorted alphabetically within the subspecies (ssp.) *aus*, *indica* and *japonica*.

Cultivar	ssp.	Mercator	Blastx	Blastp	PFAM	GO	No Annotation	Homologs WS
Dular	*aus*	61.60	65.17	59.57	59.81	37.98	27.60	90.54
N22		68.40	72.05	68.43	70.01	45.52	19.24	91.33
Anjali	*indica*	65.77	69.46	65.43	66.90	43.06	22.03	89.82
IR62266-46-6-2		68.08	71.53	67.16	68.64	44.85	20.19	91.19
IR64		67.78	71.27	67.37	69.55	45.31	20.23	82.03
IR72		63.55	67.20	62.26	63.78	41.22	24.96	88.54
CT9993-5-10-1M	*japonica*	68.57	71.80	67.58	69.24	45.01	19.62	92.43
M202		67.78	71.08	66.69	67.97	44.44	20.68	90.71
Moroberekan		65.72	69.03	64.66	66.85	43.42	22.37	91.68
Nipponbare		71.25	74.35	70.26	72.16	47.59	16.81	91.31

## Supplementary Materials

Supplementary Materials can be found at https://www.mdpi.com/1422-0067/21/21/8148/s1.

## Abbreviations

bp	Basepairs
BUSCO	Benchmarking Universal Single-Copy Orthologs
FLNC	Full-Length Non-Chimeric
GB	Gigabases
HNT	High Night Temperature
HQ	High Quality
InDel	Insertion/Deletion
IRGSP	International Rice Genome Sequencing Project
IsoSeq	Isoform Sequencing
LQ	Low Quality
ORF	Open Reading Frame
RNA-Seq	RNA Sequencing
SMRT	Single-Molecule, Real-Time
SNP	Single Nucleotide Polymorphism
